# Paedomorphic Facial Expressions Give Dogs a Selective Advantage

**DOI:** 10.1371/journal.pone.0082686

**Published:** 2013-12-26

**Authors:** Bridget M. Waller, Kate Peirce, Cátia C. Caeiro, Linda Scheider, Anne M. Burrows, Sandra McCune, Juliane Kaminski

**Affiliations:** 1 Centre for Comparative and Evolutionary Psychology, University of Portsmouth, Portsmouth, Hampshire, United Kingdom; 2 Department of Psychology, Freie Universität Berlin, Berlin, Germany; 3 Department of Physical Therapy, Duquesne University, Pittsburgh, United States of America; 4 Department of Anthropology, University of Pittsburgh, Pittsburgh, United States of America; 5 WALTHAM®, Centre for Pet Nutrition, Leicestershire, United Kingdom; University of Sydney, Australia

## Abstract

How wolves were first domesticated is unknown. One hypothesis suggests that wolves underwent a process of self-domestication by tolerating human presence and taking advantage of scavenging possibilities. The puppy-like physical and behavioural traits seen in dogs are thought to have evolved later, as a byproduct of selection against aggression. Using speed of selection from rehoming shelters as a proxy for artificial selection, we tested whether paedomorphic features give dogs a selective advantage in their current environment. Dogs who exhibited facial expressions that enhance their neonatal appearance were preferentially selected by humans. Thus, early domestication of wolves may have occurred not only as wolf populations became tamer, but also as they exploited human preferences for paedomorphic characteristics. These findings, therefore, add to our understanding of early dog domestication as a complex co-evolutionary process.

## Introduction

Wolves were domesticated early in the history of human civilization [1], and have since evolved into dogs whose lives are now inextricably linked to those of humans. The initial steps that led to wolves becoming domesticated, however, is unknown. One hypothesis suggests that wolves underwent a process of self-domestication as tamer individuals took advantage of opportunities to scavenge from human settlements during the agricultural revolution [2]. In support of this theory is recent evidence that domestic dogs exhibit genetic mutations to a starch-rich diet [3]. During domestication, dogs have departed from wolves on various other behavioral and physical dimensions [2], [4], [5], one of the most striking being paedomorphism. In many ways dogs appear more like wolf puppies than wolf adults. These features are thought to have evolved as a byproduct of the domestication process, and arose accidently when aggression was actively selected against [6], [7], for a detailed review see [8].

**Figure 1:**
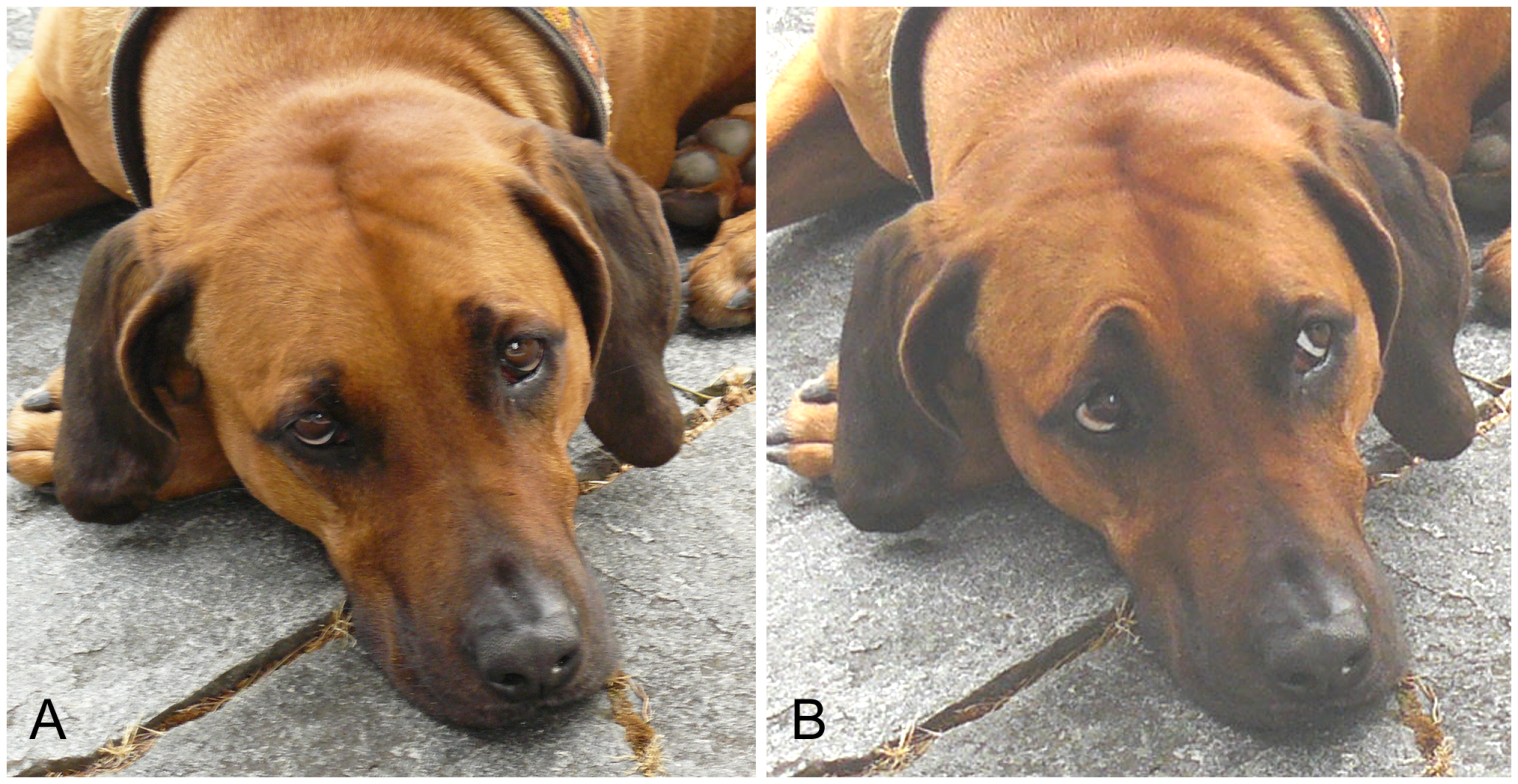
Example of facial movement AU101 (inner brow raiser) in a domestic dog (Rhodesian Ridgeback, not a subject in the study), increasing the height and overall size of the orbital cavity (eye): A) neutral on right side of face, B) AU101 on right side of face.

Paedomorphic features, however, could have evolved much earlier in response to human preferences. Domestic cats have developed modified purr vocalizations that appear to solicit increased care from human hosts by mimicking human infant cries [9], and which may have increased tolerance of cats in human environments during domestication. Likewise, the shorter snout and wider cranium of the dog give the dog face a more puppy like appearance (although there is variation between breeds) which may have evolved as the well documented human preference for paedomorphic facial characteristics [10] was exploited. Paedomorphic facial features can be further enhanced through use of upper face facial muscle contractions that lift the brow to increase the apparent height and overall size of the orbital cavity (i.e. the apparent size of the eyes: figure 1). Large eyes relative to the rest of the face are a prominent feature in human infants and are associated with perceived cuteness of and motivation to invest in human infants by human adults [10], [11]. Toys (teddy bears) that display this trait are also preferred [12], [13]. Infantile facial features are similarly preferred in pet dogs and cats [14], and manipulation of infant-like facial traits increases perceived cuteness [15]. However, in all of these studies humans are making forced choices in experimental conditions. In addition, demonstrating visual preference does not necessarily mean that these animals are (or have been) selected preferentially. To demonstrate whether these human preferences translate into differential investment we need to examine which dog characteristics incur a current selective advantage. Current fitness is not necessarily indicative of past selection of course, but it is a common assumption in behavioural ecology and evolutionary anthropology.

Juvenile traits other than face may have also been subject to selection, of course. Tail wagging and other submissive behaviours are more common in wolf puppies than adult wolves but persist in the adult dog [5], and are more often human directed [16]. Such behaviours, however, are not human-like or even universally mammalian, so it is unlikely that they would be as salient as the face (to humans), which is widely understood to be an attention grabbing stimulus in both humans and other animals.

Dog facial expressions have been described in classic studies [17], but as the facial muscles of social mammals (including humans) can exhibit great subtlety of movement, standardised methods for facial movement measurement are needed to make accurate observations within and between species. Scientists must use validated, anatomically based systems for recording facial expression. First, facial expressions are processed as whole units in an automatic, streamlined manner which makes it difficult to see the detail accurately [18]. Second, human observers tend to categorise facial expressions in terms of emotion, which can affect how comparisons between species are made [19]. The Facial Action Coding System (FACS: [20]) is an anatomically based facial expression coding system used in humans to counter these problems, which identifies observable facial changes associated with underlying muscle movement. Recently, the system has been successfully modified for use with chimpanzees [21], rhesus macaques [22], hylobatids [23] and orangutans [24]. The systems are objective, reliable and standardised, and allow subtle movements to be identified and quantified.

In the current study we used shelter dog rehoming as a proxy for dogs’ selection over time. We tested whether humans (when adopting dogs from a shelter) actively select for dogs, which appear more juvenile in the face as a result of facial muscle contraction. AU101 (inner brow raiser) raises the medial portion of the brow increasing the apparent size of the eyes in relation to the face, and as such enhances one of the features of the face associated with infants. Subtle facial muscle movements were recorded using an anatomically based facial muscle coding system (DogFACS). We examined whether frequent use of these movements (AU101: inner brow raiser) was associated with selection by humans using real world shelter dog adoption speed as a proxy for human selection over evolutionary time.

## Materials and Methods

### Ethics Statement

This study was carried out in strict accordance with the recommendations in the ASAB/ABS guidelines for the use of animals in research and was approved by the University of Portsmouth Animal Ethics Committee.

### Development of DogFACS

Footage from 28 privately owned dogs of varying breeds (approximately 8–10 hours) from the Max Planck Institute for Evolutionary Anthropology DogLab was the primary source for DogFACS development. In addition, we sourced approximately 100 clips from www.youtube.com (permission granted from the copyright holder of each clip) and used ad hoc footage from 86 dogs at four dog shelters (Portsmouth City Dog Kennels; Wood Green, The Animal’s Charity in Cambridge; The Dog’s Trust, West London, Harefield and RSPCA Southridge Animal Centre, London). Each facial movement was documented by appearance changes, minimal criteria for identification and comparison to other species, in line with FACS terminology (Table 1). The muscular basis of each facial movement was verified in light of dissection of a face from a specimen of a domestic dog (AMB) as well as previously published dissections [25]. The manual is freely available and requires certification to use (www.dogfacs.com).

**Table 1:** Comparison of action units (AUs) and the underlying facial muscles in humans [20] and dogs.

Action Units		Facial Musculature	
Humans	Dogs	Humans	Dogs
**Upper Face**			
**1** Inner brow raiser	**101** Inner brow raiser	Frontalis (medial)	Frontalis is present but it does not seem to raise the brow region. Levator anguli occuli medialis raises the inner brow region.
**2** Outer brow raiser	Not observed	Frontalis (lateral)	(As above)
**4** Brow lowerer	Not observed	Procerus, corrugator supercilii,depressor supercilii	Not present
**5** Upper lid raiser	Not observed	Levator palpebrae superioris	Not described
**6** Cheek raiser	Observed only with 143 and 145	Orbicularis occuli	Present
**7** Lid tightener	Not observed	(As above)	(As above)
**43** Eye closure	**143** Eye closure	Relaxation of levator palpebraesuperioris	Orbicularis occuli
**45** Blink	**145** Blink	(As above)	(As above)
**Lower Face**			
**9** Nose wrinkler	**109+110** Nose wrinkler and upper lipraiser - nose wrinkler hard to codeindependently	Levator labii superiorisalaeque nasi	Levator nasolabialis, caninus, levator labii maxillaris
**10** Upper lip raiser	**110** Upper lip raiser	Levator labii superioris	(As above)
**11** Nasiolabial furrow deepener	Not observed	Zygomaticus minor	Not present
**12** Lip corner puller	**12** Lip corner puller	Zygomaticus major	Zygomaticus
**13** Sharp lip puller	Not observed	Caninus	Present
**14** Dimpler	Not observed	Buccinator	Present
**15** Lip corner depressor	Not observed	Depressor anguli oris	Not present
**16** Lower lip depressor	**116** Lower lip depressor	Depressor labii inferioris	Platysma
**17** Chin raiser	Not observed	Mentalis	Present
**18** Lip pucker	**118** Lip pucker	Incisivii labii (superioris andinferioris), orbicularis oris	Only orbicularis oris present
**20** Lip stretcher	Not observed	Risorius	Not present
**22** Lip funneler	Not observed	Orbicularis oris	Present
**23** Lip tightener	Not observed	Platysma	Present
**24** Lip presser	Not observed	Orbicularis oris	Present
**25** Lips part	**25** Lips part	Orbicularis oris, depressor labiiinferioris, levator labii superioris	Orbicularis oris, caninus, levator labii maxillaris, levator nasolabialis, platysma
**26** Jaw drop	**26** Jaw drop	Non-mimetic muscles: masseter, temporalis, pterygoid and digastricus	
**27** Mouth stretch	**27** Mouth stretch	(As above)	
**Action Units**		**Facial Musculature**	
**Humans**	**Dogs**	**Humans**	**Dogs**
**Miscellaneous Action Units**			
**8** Lips towards each other	Not observed	Orbicularis oris	Present
**21** Neck tightener	Not observed	Platysma	Present
**38** Nostril dilator	Observed during sniff (AD40)	Nasalis	Not present
**39** Nostril compressor	(As above)	(As above)	(As above)

### Shelter Dog Data Collection

The study used a correlational design using data from a one-shot, timed observation. Dogs were observed at the same four re-homing shelters (above). The modal breed group (bull breeds, which includes all breeds derived from the molasser breed, Staffordshire Bull Terriers, Mastiffs and mixed bull breeds: as classified by the shelter staff using criteria from the UK Kennel Club) was chosen for analysis to minimise the variance associated with breed differences, and totalled 29 dogs. Each dog was filmed for a 2 min period (focal sampling) during controlled first contact with the experimenter. The experimenter approached the subject’s kennel room and stood in front of the room with a neutral stance and holding out one hand. Each 2 min video sample (from each dog subject) was coded using DogFACS to record the frequency of facial movements (full DogFACS coding), duration of tail wagging and time spent at the front of the kennel in close proximity to the experimenter. The number of days between becoming available for re-homing and leaving the shelter was recorded. Reliability assessment was conducted on the behavioural coding (DogFACS AUs and other behaviours: Table 2) for 30% of the sample (8 dogs) using Wexler’s Agreement (Ekman et al., 2002):
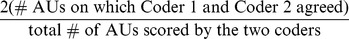



**Table 2:** Wexler’s agreement calculations for the behavioural coding.

Behaviour	Agreement
EAD101 (ears forward)	0.69
EAD102 (ears adductor)	0.79
EAD103 (ears flattener)	0.73
EAD104 (ears rotator)	0.83
AU101 (inner brow raiser)	0.78
AD19 (tongue show)	0.71
AD137 (nose wipe)	0.86
AU25 (lips parted)	0.91
AU26 (jaw drop)	0.88
Proximity	1.00
Tail wagging	0.76

Proximity and tail wagging were treated as categorical variables by using number of bouts instead of overall duration for the reliability and agreement was also assessed using Wexler’s Agreement.

## Results

**Figure 2:**
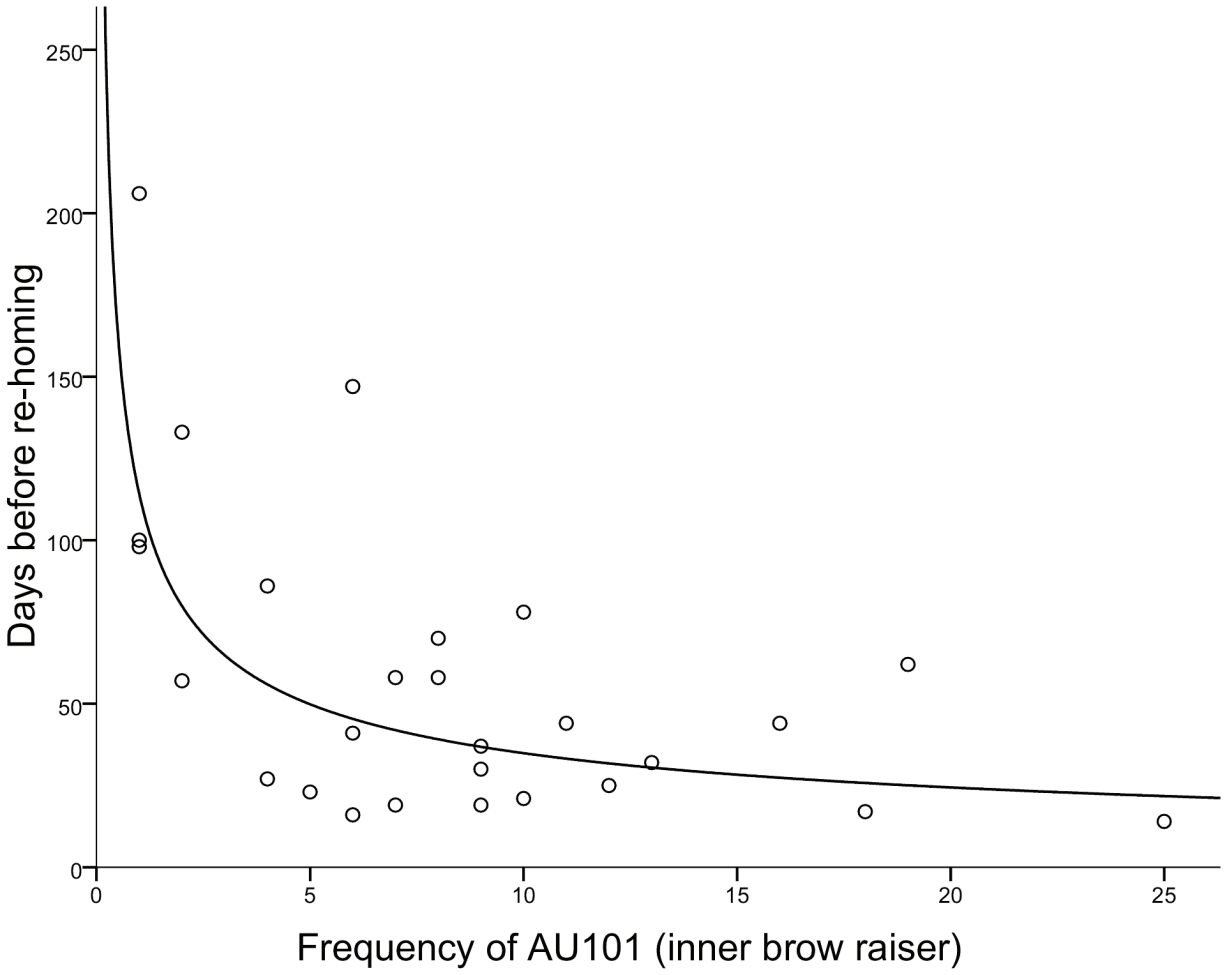
Relationship between frequency of AU101 and days before re-homing in the dog shelter. Curved line shows the power estimation.

Two dogs were removed as their time before re-homing was greater than the upper quartile by more than 1.5 IQR (82+87 days), and thus were perceived to be outliers (and their long stay most likely due to unusual factors). Our final sample included 27 dogs for analysis (Age range = 7–96 months, *M* = 29.46 months). Nonparametric correlations (as the dependent variable was not normally distributed) were used to explore the relationships between the behavioural variables and the number of days before re-homing (Table 3). AU101 and time at the front of the kennel were the only variables significantly negatively correlated with days before re-homing, indicating that dogs that produced more of these behaviours were re-homed quicker. Tail wagging was positively correlated indicating that dogs that tail wagged more were re-homed slower. Note, however, that if Bonferroni corrections were applied, no variables would be deemed significant so these exploratory findings should be taken with caution. Visual inspection of scatter plots and curve estimation were used to explore the relationships further. Time spent at the front of the kennel and tail wagging had very weak or no linear or curvilinear relationships with the dependent variable. AU101 had a significant power curve relationship with the dependent variable and the model explained a significant proportion of the variance in re-homing speed (R^2^ = 0.39, F(1,25) = 15.63, p<0.005), see Figure 2. From the regression equation (y = 114.12x^−0.515^, see Table 4) we can predict that a dog that produces five AU101 during the 2 min observation will stay in the shelter for 49.83 days on average, but if it produces 10 AU101, this would be reduced to 34.88 days, and if it produces 15, this would be reduced to 28.31 days. As there is a negative power relationship the slope becomes less steep as AU101 increases, and so the benefit (in terms of re-homing) in producing AU101 reduces with increasing AU101.

**Table 3:** Relationship between behaviours exhibited during the 2-homing.

Behaviour	Days before re-homing	
	Spearman’s rho	p value
# AU101 (inner brow raise)	−.501	.008
# AU19 (tongue show)	.070	.729
# AD137 (nose wipe)	.339	.083
# AU25 (lips parted)	.262	.187
# AU26 (jaw drop)	.268	.176
# EAD101 (ears forward)	−.331	.091
# EAD102 (ears adductor)	−.236	.236
# EAD103 (ears flattener)	−.187	.349
# EAD104 (ears rotator)	−.005	.981
Tail wagging duration	.424	.027
Time at front of kennel	−.393	.042
Age (months)^1^	.153	.474

= 24 as age was unavailable for some dogs.^1^ N

**Table 4:** Regression statistics (power curve fit) between AU101 and the number of days before re-homing, showing unstandardised co-efficients (B) and the associated standard error (SE B), standardised co-efficients (β) and significance values (P).

	B	SE B	β	P
(Constant)	114.12	30.26		
AU101	−.52	.13	−.62	.001

## Discussion

Domestic dogs who produced a high frequency of facial movement to raise the inner brow (AU101) were adopted more quickly from re-homing shelters. As AU101 enhances a key feature of paedomorphism (eye size and height: [10]) this suggests that dogs have evolved to manipulate the human preference for paedomorphic features using the face. This is the first empirical evidence that paedomorphism plays a key role in humans’ current selection of dogs, and the first time that actual investment has been used as an indicator of preference. If the selection process in the shelter context emulates past selection during domestic dog evolution, this preference may have also been at work during early dog domestication.

Interestingly, tail wagging and close proximity to the human were not strongly associated with speed of selection by adopters, despite being factors that are commonly believed to indicate a friendly temperament. In fact, higher durations of tail wagging resulted in a longer period before re-homing. This finding further supports the growing evidence that indirect manipulation of human sensory preferences (particularly a preference for juvenile facial characteristics) has been a particularly powerful selective force in domestication [2], [9], even more so than genuine indicators of temperament. Importantly, it is highly possible that these facial expressions do not correlate with suitability as a pet, but, like superficial morphological traits, are still preferred over more relevant behavioural traits [26].

In humans, the equivalent facial movement to AU101 is AU1(inner brow raiser), which features heavily in human sadness expressions [20]. It is possible, therefore, that human adopters were responding not to paedomorphism, but instead to perceived sadness in the dogs looking for adoption. However, it is also possible that the human sadness expression is itself derived from paedomorphism, and that sadness is attributed to this specific facial movement *because* it enhances paedomorphism and thus perceived vulnerability. Another possibility is that humans are responding to the increase in white sclera exposed in the dogs as the orbital cavity is stretched through AU101 action. Visibile sclera is a largely unique human trait [27] (which likely contributes to our extensive gaze following abilities) and people are more likely to cooperate or behave altruistically when exposed to cues of being watched [28], [29]. It is unclear, however, whether it is the sclera specifically or simply the presence of eyes per se which has such a powerful affect on human behavior and attention, and so this is more a complimentary hypotheses as opposed to an alternative.

Our real world data show that domestic dogs who exhibit paedomorphic characteristics are preferentially and actively selected by humans as pets from rehoming shelters. This therefore supports the hypothesis that paedomorphic characteristics in domestic dogs arose as a result of indirect selection by humans rather than only being a by-product of selection against aggression. Whether our findings are transferable to other contexts, such as breeding, is unknown, and it is possible that modern breeding practices put emphasis on such specific morphological and behavioural traits that this effect is obscured. However, given that recent evidence leans towards early wolf domestication arising from tolerance of their presence rather than direct selection per se [2], [3], adoption from shelters might be a more appropriate proxy than modern breeding. We can therefore speculate that early domestication of wolves may have occurred not only as wolf populations became tamer [2], [3], but also as they exploited human preferences for paedomorphic characteristics.

## Supporting Information

Raw Data S1 **The raw data.**
(SAV)
